# Prevalence of caffeine consumers, daily caffeine consumption, and factors associated with caffeine use among active duty United States military personnel

**DOI:** 10.1186/s12937-022-00774-0

**Published:** 2022-04-14

**Authors:** Joseph J. Knapik, Ryan A. Steelman, Daniel W. Trone, Emily K. Farina, Harris R. Lieberman

**Affiliations:** 1 Military Nutrition Division, US Army Research Institute of Environmental Medicine, 10 General Greene Ave, Natick, MA 01760 USA; 2 US Army Public Health Center, 8252 Blackhawk Road, Aberdeen Proving Ground, MD 21010 USA; 3 Naval Health Research Center, Building 329, Ryne Road, San Diego, CA 92152 USA

**Keywords:** Coffee, Tea, Soda, Energy drinks

## Abstract

**Background:**

Although representative data on caffeine intake in Americans are available, these data do not include US service members (SMs). The few previous investigations in military personnel largely involve convenience samples. This cross-sectional study examined prevalence of caffeine consumers, daily caffeine consumption, and factors associated with caffeine use among United States active duty military service members (SMs).

**Methods:**

A stratified random sample of SMs were asked to complete an on-line questionnaire on their personal characteristics and consumption of caffeinated products (exclusive of dietary supplements). Eighteen percent (*n* = 26,680) of successfully contacted SMs (*n* = 146,365) completed the questionnaire.

**Results:**

Overall, 87% reported consuming caffeinated products ≥1 time/week. Mean ± standard error per-capita consumption (all participants) was 218 ± 2 and 167 ± 3 mg/day for men and women, respectively. Caffeine consumers ingested 243 ± 2 mg/day (251 ± 2 mg/day men, 195 ± 3 mg/day women). On a body-weight basis, men and women consumed respectively similar caffeine amounts (2.93 vs 2.85 mg/day/kg; *p* = 0.12). Among individual caffeinated products, coffee had the highest use (68%), followed by sodas (42%), teas (29%), energy drinks (29%) and gums/candy/medications (4%). In multivariable logistic regression, characteristics independently associated with caffeine use (≥1 time/week) included female gender, older age, white race/ethnicity, higher body mass index, tobacco use or former use, greater alcohol intake, and higher enlisted or officer rank.

**Conclusion:**

Compared to National Health and Nutrition Examination Survey data, daily caffeine consumption (mg/day) by SMs was higher, perhaps reflecting higher mental and physical occupational demands on SMs.

## Background

Caffeine is a widely consumed psychoactive stimulant ingested in various beverages including coffees, teas, sodas, and energy drinks. About 90% of United States (US) adults consume caffeinated products with little difference between men and women in how frequently the products are ingested [1, 2]. Among caffeine consumers, the average caffeine intake is about 211 mg/day [1]. Although there are cases where consumption of very high dosages of caffeine has led to seizures, transient cardiovascular problems, and even deaths [3, 4], comprehensive reviews have concluded that consumption of < 400 mg/day is generally safe, enhances certain aspects of mental, physical, and occupational performance, and may confer other health benefits [5–7]. Among healthy adults, moderate coffee consumption has been reported to be associated with reduced risk of certain health conditions including chronic liver disease, gout, Parkinson’s disease, Alzheimer’s disease, Type 2 diabetes, certain types of cancers, all-cause mortality, and cause-specific mortality [5, 8, 9]. However, concerns about caffeine use by pregnant women and increased consumption of energy drinks by young adults has been expressed [5, 10]. For pregnant women, caffeine dosages in the range of < 200 to 300 mg/day are recommended [7] because of increased risk for pregnancy-related adverse effects (low birth weight, pregnancy loss, childhood leukemia) [8]. Data from the nationally-representative National Health and Nutrition Examination Survey (NHANES) indicate caffeine consumption from energy drinks increased from 2001 to 2016 in 19- to 39-year-olds, but was relatively low [10, 11]. Furthermore, total caffeine consumption did not significantly increase, as there was a concurrent reduction in caffeine intake from sodas in 2001–2010 [11].

Although representative data on caffeine intake in Americans are available [1, 2, 11–15], these data do not include US service members (SMs). Among civilians, caffeine use varies depending on occupation; working in “legal” and “management” occupations was associated with greater caffeine intake than the average of all other occupations investigated [15]. Military personnel must engage in a number of physically- and cognitively-demanding tasks such as intelligence gathering, tactical planning, guard duty, lengthy marches with heavy backpacks, lifting and carrying substantial loads, movement over and under obstacles, and other operational tasks that can require lengthy and intense activity. Furthermore, military work hours are not regulated (restricted) by the labor laws governing the civilian population in the US, and active duty SMs are employed full-time. The extensive physical demands placed on SMs include early morning physical training and limited sleep during training, operations, and deployments. Studies of US SMs have found that approximately 70% sleep < 6 h per night and only ~ 30% are getting the recommended 7–8 h of sleep per night [16]. This contrasts with studies of the US civilian population where 72% of civilians sleep ≥7 h per night and only 28% sleep < 6 h/night [16, 17]. Demanding tasks and lack of adequate sleep may lead SMs to consume more caffeinated substances than the general population. In particularly demanding circumstances, such as combat environments, and in populations such as aviators, caffeine use is particularly high [18, 19].

Caffeine consumption in Army [20], Navy/Marine Corps [21] and Air Force [22] personnel has been investigated in separate surveys by our group, usually in convenience samples, and was higher than the civilian population [1, 2, 15, 20–22]. The purpose of the current investigation was to examine the more recent prevalence of caffeine consumers, amount of caffeine consumption, and factors associated with use in a single, large, stratified random sample of US military personnel from all services.

## Materials and methods

This investigation involved a cross-sectional survey completed by US military SMs and was part of a larger study involving the health effects of dietary supplements [23]. The Naval Health Research Center’s institutional review board approved the study protocol, and SMs consented to participate by signing an informed consent document. Investigators adhered to policies and procedures for protection of human subjects as prescribed by Department of Defense Instruction 3216.01, and the research was conducted in adherence with provisions of Title 32, Code of Federal Regulations, Part 219.

### Sampling frame and solicitation procedures

Details of the sampling frame, solicitation of SMs, participant flow through the study, and response bias were described previously [23]. Briefly, investigators requested a random sample of 200,000 SMs, stratified by sex (88% male and 12% female) and branch of service (Army 36%, Air Force 24%, Marine Corps 15%, and Navy 25%), from the Defense Manpower Data Center. Recruitment of SMs into the study from this random sample involved a maximum of 12 sequential contacts. Investigators sent the prospective participant an introductory postal letter with a $1 pre-incentive designed to increase the response rate [24, 25]. The letter described the survey, included a link to a secure website, and a unique login that could be used to access the web-based survey and electronically sign the consent form. SMs who did not initially complete the survey were sent a follow-up email message after 10 days, and a postcard after 3 weeks as a reminder. If the SM did not respond after having received the postcard, he/she received up to seven additional email reminders and three postcards evenly distributed during the time the survey was open. Reminders were sent only to those who had not responded. All postal and on-line contacts stated the SM could decline participation at any time and be removed from the contact list. Recruitment began in December 2018; after August 2019, no further recruitment was conducted, and no surveys were accepted.

### Survey description

The questionnaire was administered on-line and was designed to characterize participants and quantify the frequency and amount of caffeinated products they consumed in the last 6 months. To characterize participants, there were questions on demographics (gender, age, education, race/ethnicity, height, weight), lifestyle factors (exercise, tobacco use, alcohol consumption, sleep), and military characteristics (rank, occupation assignment, service branch). Participants were asked to select serving sizes for coffee, tea, and soft drinks and how often they were consumed. Sizes available for selection included 8, 12, 16, 20, and 24+ ounces; frequencies available for selection included per day, week, month, or year; and number of times consumed available for selection ranged from 0 to 8 for each frequency option. For energy shots and energy beverages, participants provided the number of bottles or packets (0 to 16) and the frequency (per day, week, month, or year). For caffeinated candy and gum, participants provided the number of candies or sticks of gum (0 to 16) and the frequency (per day, week, month, or year). For caffeinated medications, participants provided the number of pills consumed (0 to 16) and the frequency (per day, week, month, or year). Examples were provided for energy shots (e.g. 5-Hour Energy), energy beverages (e.g., Red Bull, Monster, Full Throttle), caffeinated candy/gum (e.g., Military Energy, GoFastGum), and caffeinated medications (e.g., Bayer, Excedrin, No Doz).

### Statistical analysis

Statistical analyses were conducted using the Statistical Package for the Social Sciences (Version 21.0.0.0, 2019, IBM Corporation). Caffeine products were grouped into five types: 1) coffee; 2) tea; 3) sodas; 4) energy drinks (energy shots and energy beverages combined); 5) caffeinated gums/medications (candies, gum, and medications combined). All types were then combined to determine an aggregated caffeine intake (i.e., any caffeine product).

An individual who used any caffeinated product ≥1 time/week was considered a caffeine consumer. This frequency was selected to be relatively consistent with consumption frequencies used in other studies [2, 12, 20–22, 26–28]. Caffeine consumption (mg/day) was calculated based on the serving size and the frequency of consumption using publicly available databases and estimates of caffeine content in various products [27, 29]. One ounce of coffee, tea, and soda was considered to contain 12, 6, and 3 mg caffeine per ounce, respectively. Energy drinks and energy shots were considered to contain 160 and 200 mg caffeine, respectively. Energy gum/candy and medication were considered to contain 50 and 100 mg caffeine, respectively.

Body mass index (BMI) was calculated as self-reported weight/height^2^ (kg/m^2^). Weekly duration of aerobic training or resistance training (minutes/week) was calculated by multiplying weekly exercise frequency (sessions/week) by the duration of training (minutes/session). Tobacco users were defined as individuals who reported using any tobacco products in the past week; former tobacco users were defined as those who reported having used tobacco products in the past but had quit within the last year or earlier.

“Caffeine prevalence” refers to the proportion (%) of SMs using a caffeinated product ≥1 day/week; “caffeine consumption” refers the total amount of caffeine consumed each day (mg/day). Prevalence of caffeine consumers (%) with standard errors (SE) was calculated for each caffeine product type individually and for all caffeine products in aggregate (i.e., any caffeine). Chi-square statistics were used to examine differences in the prevalence of caffeine consumers across various strata of demographic, lifestyle, and military characteristics. A one-way analysis of variance (ANOVA) examined differences in caffeine consumption (mg/day) across strata of these characteristics. For ordinal variables (i.e., age, education, BMI, aerobic training duration, resistance training duration, alcohol intake, sleep), tests for linear trend, Mantel-Haenszel statistics, and ANOVA linear contrasts were also performed. Since some participants did not complete all of the questions, tables present the number of participants for each variable. Multivariable logistic regression was used to examine associations between use and non-use of caffeine products (≥1 time/week) and independent variables that included all the demographic, lifestyle, and military characteristics. Six separate regression models were developed for each caffeine product type: “any caffeine,” coffee, tea, soda, energy drinks, and caffeinated gum/medications. A one-way ANOVA for linear trend compared caffeine consumption across age groups in men and women separately. Self-reported sleep duration was not included in the multivariable analyses because only 78% of SMs responded to this question. Since multivariable analysis requires complete data on all variables, including sleep duration would have removed a large number of SMs from the multivariable analyses.

A separate analysis compared high versus lower caffeine consumers, defined as ≥400 mg/day and < 400 mg/day, respectively. Prevalence of caffeine consumers (%) with SEs were calculated for high and lower consumers on each of the demographic, lifestyle, and military characteristic. Univariable logistic regression compared unadjusted differences between high and low consumers across the characteristics; multivariable logistic regression compared differences adjusted for all demographic, lifestyle and military characteristics.

## Results

From the initial list of 200,000 potential volunteers, 146,365 (73%) were successfully contacted (i.e., no returned postal mail). Of these, 26,680 (18.2%) signed the informed consent and completed the questionnaire online.

### Caffeine use prevalence

Table 1 provides the prevalence of caffeine consumers by demographic, lifestyle, and military characteristics. Overall, 87% of participants reported using products containing caffeine ≥1 time per week, with coffee and soda being the most frequently employed. For energy drinks and energy shots considered individually, use (prevalence±SE) was 28.4 ± 0.3% and 2.4 ± 0.1%, respectively; for gums/candies and medications individually, use was 0.9 ± 0.1% and 3.5 ± 0.1%, respectively.

**Table 1:** Prevalence (% ± SE) of reported caffeine consumption (≥1 time/week) in military personnel by demographic, lifestyle, and military characteristics

Variable	Strata	Any Caffeine	Coffee	Tea	Soda	Energy Drink	Gums & Medications
Cohort	All (*n* = 26,680)	86.9 ± 0.2	68.1 ± 0.3	29.2 ± 0.3	41.9 ± 0.3	29.3 ± 0.3	4.3 ± 0.1
Gender	Men (*n* = 23,038)	87.0 ± 0.2	68.3 ± 0.3	27.0 ± 0.3	43.1 ± 0.3	31.3 ± 0.3	3.9 ± 0.1
	Women (*n* = 3642)	86.1 ± 0.6	66.9 ± 0.8	43.5 ± 0.8	33.8 ± 0.8	16.8 ± 0.6	6.5 ± 0.4
	*p*-value (chi-square)	0.13	0.10	< 0.01	< 0.01	< 0.01	< 0.01
Age	18–24 years (*n* = 4660)	77.6 ± 0.6	52.7 ± 0.7	24.9 ± 0.6	40.8 ± 0.7	34.7 ± 0.7	3.5 ± 0.3
	25–29 years (*n* = 5580)	85.1 ± 0.5	66.1 ± 0.6	28.0 ± 0.6	38.6 ± 0.7	34.2 ± 0.6	3.6 ± 0.2
	30–39 years (*n* = 11,030)	89.5 ± 0.3	72.2 ± 0.5	29.3 ± 0.4	42.7 ± 0.5	30.7 ± 0.4	4.7 ± 0.2
	≥40 years (*n* = 5275)	91.4 ± 0.4	75.1 ± 0.6	34.0 ± 0.7	44.8 ± 0.7	17.3 ± 0.5	4.8 ± 0.3
	p-value (chi square/trend)	< 0.01/< 0.01	< 0.01/< 0.01	< 0.01/< 0.01	< 0.01/< 0.01	< 0.01/< 0.01	< 0.01/< 0.01
Formal Education	Some high school/High school graduate (*n* = 3879)	79.9 ± 0.6	55.0 ± 0.8	22.9 ± 0.7	45.0 ± 0.8	38.8 ± 0.8	4.1 ± 0.3
	Some college/Associate’s degree (*n* = 11,378)	86.4 ± 0.3	67.1 ± 0.4	28.2 ± 0.4	43.1 ± 0.5	35.1 ± 0.5	4.8 ± 0.2
	Bachelor’s/Graduate degree (*n* = 11,417)	89.6 ± 0.3	73.5 ± 0.4	32.3 ± 0.4	39.6 ± 0.5	20.3 ± 0.4	3.8 ± 0.2
	p-value (chi square/trend)	< 0.01/< 0.01	< 0.01/< 0.01	< 0.01/< 0.01	< 0.01/< 0.01	< 0.01/< 0.01	< 0.01/0.06
Race / Ethnicity	White (*n* = 16,316)	90.9 ± 0.2	73.0 ± 0.3	29.0 ± 0.4	44.4 ± 0.4	31.4 ± 0.4	4.5 ± 0.2
	Hispanic (*n* = 4227)	83.5 ± 0.6	65.2 ± 0.7	25.2 ± 0.8	39.2 ± 0.8	30.4 ± 0.7	3.6 ± 0.3
	Black (*n* = 2966)	72.2 ± 0.8	46.5 ± 0.9	30.7 ± 0.8	34.4 ± 0.9	18.8 ± 0.7	4.6 ± 0.4
	Other (*n* = 3171)	84.5 ± 0.6	67.1 ± 0.8	34.6 ± 0.8	39.4 ± 0.9	27.1 ± 0.8	3.7 ± 0.3
	*p*-value (chi-square)	< 0.01	< 0.01	< 0.01	< 0.01	< 0.01	0.03
Body mass index	< 25.0 kg/m^2^ (*n* = 7856)	84.4 ± 0.4	64.9 ± 0.5	30.6 ± 0.5	39.4 ± 0.6	24.7 ± 0.5	3.5 ± 0.2
	25.0–29.9 kg/m^2^ (*n* = 13,897)	87.5 ± 0.3	69.4 ± 0.4	28.3 ± 0.4	41.2 ± 0.4	30.3 ± 0.4	4.2 ± 0.2
	≥30.0 kg/m^2^ (*n* = 4424)	89.4 ± 0.5	69.6 ± 0.7	29.3 ± 0.7	48.4 ± 0.8	34.3 ± 0.7	5.9 ± 0.3
	*p*-value (chi square/trend)	< 0.01/< 0.01	< 0.01/< 0.01	< 0.01/0.30	< 0.01/< 0.01	< 0.01/< 0.01	< 0.01/< 0.01
Aerobic exercise duration	≤90 min/wk. (*n* = 7286)	84.0 ± 0.4	66.5 ± 0.6	27.1 ± 0.5	42.5 ± 0.6	28.3 ± 0.5	4.3 ± 0.2
	91–180 min/wk. (*n* = 7285)	90.0 ± 0.4	71.4 ± 0.5	31.1 ± 0.5	45.7 ± 0.6	28.9 ± 0.5	4.2 ± 0.2
	181–300 min/wk. (*n* = 5869)	88.9 ± 0.4	70.5 ± 0.6	30.1 ± 0.6	42.4 ± 0.6	30.1 ± 0.6	4.0 ± 0.3
	≥301 min/wk. (*n* = 6240)	84.9 + 0.5	65.6 ± 0.6	28.7 ± 0.6	36.2 ± 0.6	30.4 ± 0.6	4.5 ± 0.3
	*p*-value (chi square/trend)	< 0.01/0.49	< 0.01/0.62	< 0.01/0.09	< 0.01/< 0.01	0.03/< 0.01	0.57/0.64
Resistance training frequency	≤45 min/wk. (*n* = 7776)	85.5 ± 0.4	66.4 ± 0.5	30.9 ± 0.5	47.4 ± 0.6	24.2 ± 0.5	4.5 ± 0.2
	46–135 min/wk. (*n* = 6257)	90.1 ± 0.4	71.7 ± 0.6	32.1 ± 0.6	46.0 ± 0.6	26.7 ± 0.6	4.3 ± 0.3
	136–300 min/wk. (*n* = 6581)	89.0 ± 0.4	71.3 ± 0.6	29.3 ± 0.6	39.5 ± 0.6	31.1 ± 0.6	4.2 ± 0.3
	≥301 min/wk. (*n* = 6066)	82.9 ± 0.5	63.1 ± 0.6	24.0 ± 0.5	33.0 ± 0.6	36.8 ± 0.6	4.0 ± 0.3
	*p*-value (chi square/trend)	< 0.01/< 0.01	< 0.01/< 0.01	< 0.01/< 0.01	< 0.01/< 0.01	< 0.01/< 0.01	0.46/0.11
Smoking	Never (*n* = 16,706)	84.9 ± 0.3	63.7 ± 0.4	29.7 ± 0.4	39.5 ± 0.4	24.5 ± 0.3	4.0 ± 0.2
	Former (*n* = 4767)	93.9 ± 0.3	79.9 ± 0.6	29.4 ± 0.7	44.1 ± 0.7	35.0 ± 0.7	5.2 ± 0.3
	Smoker (*n* = 4511)	93.6 ± 0.4	77.1 ± 0.6	29.9 ± 0.7	51.7 ± 0.7	43.6 ± 0.7	4.8 ± 0.3
	*p*-value (chi-square)	< 0.01	< 0.01	0.88	< 0.01	< 0.01	< 0.01
Smokeless tobacco use	Never (*n* = 20,378)	86.4 ± 0.2	66.1 ± 0.3	30.6 ± 0.3	41.6 ± 0.3	26.2 ± 0.3	4.3 ± 0.1
	Former (*n* = 2047)	95.7 ± 0.4	82.9 ± 0.8	27.1 ± 1.0	43.7 ± 1.1	38.1 ± 1.1	4.8 ± 0.5
	Smoker (*n* = 3114)	94.3 ± 0.4	79.2 ± 0.7	26.4 ± 0.8	48.5 ± 0.9	47.8 ± 0.9	4.6 ± 0.4
	*p*-value (chi-square)	< 0.01	< 0.01	< 0.01	< 0.01	< 0.01	0.43
Alcohol intake	0 ml/wk. (*n* = 8372)	73.8 ± 0.5	50.8 ± 0.5	23.3 ± 0.5	35.1 ± 0.5	23.9 ± 0.5	3.8 ± 0.2
	0.23–24.85 ml/wk. (*n* = 6132)	89.5 ± 0.4	70.2 ± 0.6	31.4 ± 0.6	41.2 ± 0.6	25.3 ± 0.6	3.9 ± 0.2
	24.86–71.69 ml/wk. (*n* = 6108)	93.3 ± 0.3	76.0 ± 0.5	31.5 ± 0.6	45.1 ± 0.6	31.9 ± 0.6	4.4 ± 0.3
	≥71.70 ml/wk. (*n* = 6067)	95.9 ± 0.3	81.9 ± 0.5	32.9 ± 0.6	48.7 ± 0.6	38.3 ± 0.6	5.0 ± 0.3
	*p*-value (chi square/trend)	< 0.01/< 0.01	< 0.01/< 0.01	< 0.01/< 0.01	< 0.01/< 0.01	< 0.01/< 0.01	< 0.01/< 0.01
Sleep	≥7 h/night (*n* = 9702)	87.9 ± 0.3	69.3 ± 0.5	29.0 ± 0.5	40.8 ± 0.5	26.2 ± 0.4	3.3 ± 0.2
	5–6 h/night (*n* = 10,027)	90.9 ± 0.3	71.5 ± 0.5	30.6 ± 0.5	46.7 ± 0.5	34.7 ± 0.5	5.1 ± 0.2
	< 4 h/night (*n* = 1090)	84.2 ± 1.1	65.4 ± 1.4	31.6 ± 1.4	43.7 ± 1.5	35.3 ± 1.4	8.2 ± 0.8
	*p*-value (chi-square/linear trend)	< 0.01/0.01	< 0.01/0.48	0.02/< 0.01	< 0.01/< 0.01	< 0.01/< 0.01	< 0.01/< 0.01
Rank	Junior enlisted, E1-E4 (*n* = 2496)	74.9 ± 0.9	49.8 ± 1.0	24.7 ± 0.9	40.5 ± 1.0	33.1 ± 0.9	3.4 ± 0.4
	Mid enlisted E5-E6 (n = 11,609)	85.2 ± 0.3	64.5 ± 0.4	27.8 ± 0.4	42.8 ± 0.5	36.4 ± 0.4	4.7 ± 0.2
	Senior enlisted E7-E9 (*n* = 4365)	91.0 ± 0.4	74.4 ± 0.7	28.9 ± 0.7	44.7 ± 0.8	29.6 ± 0.7	4.9 ± 0.3
	Warrant officer (*n* = 576)	93.2 ± 1.0	78.1 ± 1.7	29.7 ± 1.9	39.2 ± 2.0	27.3 ± 1.9	4.7 ± 0.9
	Junior officer, O1-O3 (*n* = 3891)	88.3 ± 0.5	73.6 ± 0.7	31.9 ± 0.7	35.3 ± 0.8	21.7 ± 0.7	3.2 ± 0.3
	Senior officer, O4-O7 (*n* = 3742)	92.8 ± 0.4	77.2 ± 0.7	34.2 ± 0.8	43.7 ± 0.8	12.9 ± 0.5	3.8 ± 0.3
	*p*-value (chi-square)	< 0.01	< 0.01	< 0.01	< 0.01	< 0.01	< 0.01
Occupational assignment group	Combat arms (*n* = 6451)	89.0 ± 0.4	72.5 ± 0.6	27.7 ± 0.6	41.4 ± 0.6	31.9 ± 0.6	3.8 ± 0.2
	Combat support (*n* = 10,424)	86.3 ± 0.3	66.2 ± 0.5	29.0 ± 0.4	42.1 ± 0.5	31.1 ± 0.5	4.2 ± 0.2
	Combat service support (*n* = 9132)	86.3 ± 0.4	67.6 ± 0.5	30.6 ± 0.5	42.0 ± 0.5	25.7 ± 0.5	4.6 ± 0.2
	*p*-value (chi-square)	< 0.01	< 0.01	< 0.01	0.65	< 0.01	0.04
Service branch	Air Force (*n* = 9788)	86.3 ± 0.3	65.5 ± 0.5	29.7 ± 0.5	43.1 ± 0.5	28.3 ± 0.5	3.9 ± 0.2
	Army (*n* = 7935)	87.0 ± 0.4	69.0 ± 0.5	28.1 ± 0.5	39.8 ± 0.5	29.3 ± 0.5	4.5 ± 0.2
	Marine Corps (*n* = 3194)	84.8 ± 0.6	65.5 ± 0.8	23.8 ± 0.8	40.7 ± 0.9	38.8 ± 0.9	3.6 ± 0.3
	Navy (*n* = 5763)	88.8 ± 0.4	72.8 ± 0.6	33.0 ± 0.6	43.3 ± 0.7	25.9 ± 0.6	5.0 ± 0.3
	*p*-value (chi-square)	< 0.01	< 0.01	< 0.01	< 0.01	< 0.01	< 0.01

Table 1 indicates there was little difference between men and women in aggregate use of any caffeinated product or coffee; however, women were much more likely to use tea and gums/medications while men were much more likely to use soda and energy drinks. The proportion of SMs using any caffeinated product increased with age, especially for coffee, tea, soda, and gums/medications, but use of energy drinks decreased with age. The proportion of SMs using any caffeinated product increased with formal educational level, especially for coffee and tea, while use of soda and energy drinks decreased as formal education increased; use of gums/medications were highest among those with some college. Among race/ethnicities, white SMs had the largest proportion using caffeinated products, especially for coffee, soda, and energy drinks, while black SMs had the lowest proportion using these same products. As BMI increased, so did use of most caffeinated products, except tea, as prevalence was highest among the lowest BMI category.

For most caffeinated products, aerobic exercise duration was not related to use in any systematic way, although energy drink use increased modestly as exercise duration increased. SMs performing the most resistance training had the lowest use of any caffeinated products, especially for coffee and tea. As resistance training increased, use of soda decreased, and use of energy drinks increased. Among those who reported any weekly resistance training (*n* = 22,872), use of any caffeinated product was 87.9 ± 0.2%, compared to 80.5 ± 0.6% among those who did not report any weekly resistance training (*n* = 3808) (*p* < 0.01). Smokers and former smokers had the highest use of caffeinated products among all product types except tea, where there were no significant differences among groups. Smokeless tobacco users and former users also had the highest use of caffeine for all product types except tea, where those who had never used smokeless tobacco had the highest caffeine use. Use of caffeinated products among all types increased as alcohol consumption increased. Those reporting ≥5 h/night of sleep had the highest aggregated caffeine and coffee use, but those reporting < 6 h/night had the highest use of tea, soda, energy beverages, and gums/medications.

Among both enlisted SMs and officers, as rank increased, so did aggregated use of caffeinated products, especially coffee, tea, soda, and gum/medication. For energy drinks, the trend was the opposite: as rank increased, energy drink use decreased. Enlisted soldiers were more likely to use energy drinks than officers, and the lowest use of energy drinks was among senior officers. SMs in combat arms occupations were more likely to use any caffeinated product, especially coffee and energy drinks, while combat service support personnel had the highest use of tea and gums/medications. Navy personnel had the highest use of caffeinated products of all types, except energy drinks, where Marine Corps personnel had the highest use.

### Caffeine consumption

Table 2 provides the estimated daily caffeine consumption (mg/day) among caffeine consumers by their demographic, lifestyle, and military characteristics. The average daily consumption of caffeine was 243 mg/day. Coffee, tea, soda, energy drinks, and gums/medications accounted for 69, 8, 6, 17, and < 1% of caffeine consumption, respectively. The per-capita consumption (all participants including non-consumers) was 211 ± 1 mg/day, with men ingesting 218 ± 2 mg/day and women 167 ± 3 mg/day.

**Table 2:** Caffeine consumption (mean ± SE mg/day) of consumers (≥1 time/week) by demographic, lifestyle, and military characteristics

Variable	Strata	Any Caffeine	Coffee	Tea	Soda	Energy Drink	Gums & Medications
Group	All (*n* = 23,175)	243.1 ± 1.5	166.6 ± 1.2	18.5 ± 0.4	15.8 ± 0.2	41.0 ± 0.6	1.0 ± 0.2
Gender	Men (*n* = 20,040)	250.7 ± 1.7	171.8 ± 1.5	17.5 ± 0.4	16.5 ± 0.2	43.9 ± 0.7	1.0 ± 0.2
	Women (*n* = 3.135)	194.5 ± 3.0	133.8 ± 2.5	25.2 ± 0.9	11.5 ± 0.5	23.7 ± 1.3	1.2 ± 0.3
	*p*-value (ANOVA)	< 0.01	< 0.01	< 0.01	< 0.01	< 0.01	0.60
Age	18–24 years (*n* = 3615)	203.6 ± 4.6	108.9 ± 2.7	19.5 ± 1.0	17.4 ± 0.7	56.0 ± 2.1	1.8 ± 0.6
	25–29 years (*n* = 4746)	226.1 ± 3.1	147.7 ± 2.4	16.5 ± 0.7	13.1 ± 0.4	47.7 ± 1.5	1.0 ± 0.2
	30–39 years (*n* = 9875)	254.7 ± 2.2	179.2 ± 1.8	17.3 ± 0.5	15.5 ± 0.3	42.0 ± 0.9	0.7 ± 0.2
	≥40 years (*n* = 4821)	264.8 ± 3.4	201.6 ± 2.8	22.0 ± 0.8	18.0 ± 0.5	22.1 ± 1.1	1.1 ± 0.4
	*p*-value (ANOVA/trend)	< 0.01/< 0.01	< 0.01/< 0.01	< 0.01/0.03	< 0.01/0.07	< 0.01/0.01	0.15/0.14
Formal Education	Some high school/high school graduate (*n* = 3099)	242.3 ± 4.8	129.3 ± 1.9	20.4 ± 1.3	20.9 ± 0.8	69.7 ± 2.5	1.9 ± 0.4
	Some college/Associate’s degree (*n* = 9836)	243.3 ± 2.4	159.5 ± 1.7	18.1 ± 0.5	16.5 ± 0.4	48.3 ± 1.0	1.0 ± 0.3
	Bachelor’s/Graduate degree (*n* = 10,234)	243.0 ± 2.1	184.8 ± 1.2	18.4 ± 0.5	13.6 ± 0.3	25.4 ± 0.8	0.8 ± 02
	*p*-value (ANOVA/trend)	0.98/0.88	< 0.01/< 0.01	0.10/0.06	< 0.01/< 0.01	< 0.01/< 0.01	0.06/0.02
Race / Ethnicity	White (*n* = 14,827)	263.5 ± 1.9	184.8 ± 1.5	18.0 ± 0.4	17.3 ± 0.3	42.5 ± 0.8	0.8 ± 0.2
	Hispanic (*n* = 3530)	227.7 ± 4.1	151.8 ± 3.0	15.9 ± 0.9	13.5 ± 0.6	45.3 ± 2.0	1.1 ± 0.3
	Back (*n* = 2140)	158.8 ± 3.8	90.9 ± 2.7	21.5 ± 1.0	13.1 ± 0.7	31.1 ± 1.9	2.2 ± 0.5
	Other (*n* = 2678)	217.5 ± 3.9	146.3 ± 3.1	22.0 ± 1.1	12.6 ± 0.6	35.3 ± 1.6	1.3 ± 0.3
	*p*-value (ANOVA)	< 0.01	< 0.01	< 0.01	< 0.01	< 0.01	0.06
Body mass index	< 25.0 kg/m^2^ (*n* = 6629)	215.7 ± 3.0	145.4 ± 2.0	19.2 ± 0.7	15.1 ± 0.4	34.3 ± 1.2	1.6 ± 0.5
	25.0–29.9 kg/m^2^ (*n* = 12,163)	251.2 ± 2.0	175.7 ± 1.7	17.7 ± 0.5	15.2 ± 0.3	42.0 ± 0.9	0.7 ± 0.1
	≥30.0 kg/m^2^ (*n* = 3956)	260.5 ± 3.5	172.3 ± 2.9	19.2 ± 0.9	19.1 ± 0.6	48.8 ± 1.5	1.1 ± 0.3
	*p*-value (ANOVA/trend)	< 0.01/< 0.01	< 0.01/< 0.01	0.09/0.97	< 0.01/< 0.01	< 0.01/< 0.01	0.03/0.28
Aerobic exercise weekly duration	< 90 min/wk. (*n* = 6120)	235.7 ± 3.0	161.0 ± 2.2	17.8 ± 0.7	16.5 ± 0.4	39.2 ± 1.2	1.1 ± 0.4
	90–180 min/wk. (*n* = 6560)	236.2 ± 2.4	165.3 ± 2.0	17.6 ± 0.6	16.5 ± 0.4	36.3 ± 1.0	0.6 ± 0.1
	181–300 min/wk. (*n* = 5218)	249.1 ± 3.1	173.9 ± 2.5	17.5 ± 0.7	15.1 ± 0.4	42.0 ± 1.4	0.6 ± 0.1
	> 300 min/wk. (*n* = 5277)	254.0 ± 3.9	167.7 ± 2.8	21.5 ± 0.7	14.9 ± 0.5	48.1 ± 1.6	1.8 ± 0.5
	*p*-value (ANOVA/trend)	< 0.01/< 0.01	< 0.01/0.01	< 0.01/< 0.01	0.01/< 0.01	< 0.01/< 0.01	0.02/0.15
Resistance training weekly duration	≤45 min/wk. (*n* = 6650)	250.8 ± 3.0	172.8 ± 2.3	21.1 ± 0.7	20.6 ± 0.5	35.3 ± 1.2	1.0 ± 0.3
	46–135 min/wk. (*n* = 5636)	239.3 ± 2.6	171.2 ± 2.3	18.3 ± 0.7	15.8 ± 0.4	33.5 ± 1.0	0.6 ± 0.1
	136–300 min/wk. (*n* = 5859)	238.5 ± 2.7	166.8 ± 2.2	16.3 ± 0.6	13.5 ± 0.4	41.2 ± 1.2	0.8 ± 0.2
	≥301 min/wk. (*n* = 5030)	242.3 ± 3.9	153.2 ± 2.7	18.0 ± 0.8	12.3 ± 0.4	56.9 ± 1.6	1.8 ± 0.5
	*p*-value (ANOVA/trend)	0.01/0.06	< 0.01/0.01	< 0.01/< 0.01	< 0.01/< 0.01	< 0.01/< 0.01	0.05/0.08
Smoking	Never (*n* = 14,177)	215.5 ± 1.8	148.2 ± 1.4	18.7 ± 0.4	14.6 ± 0.3	33.0 ± 0.7	1.0 ± 0.2
	Former user (*n* = 4475)	278.8 ± 3.4	198.4 ± 2.8	17.5 ± 0.8	15.6 ± 0.5	46.3 ± 1.4	0.9 ± 0.2
	Current user (*n* = 4222)	298.7 ± 4.4	195.3 ± 3.2	19.1 ± 0.9	20.3 ± 0.6	62.7 ± 1.9	1.3 ± 0.5
	*p*-value (ANOVA)	< 0.01	< 0.01	0.34	< 0.01	< 0.01	0.70
Smokeless tobacco use	Never (*n* = 17,597)	226.8 ± 1.6	155.8 ± 1.3	18.7 ± 0.4	15.6 ± 0.3	35.8 ± 0.7	1.0 ± 0.2
	Former user (*n* = 1959)	294.5 ± 4.7	213.9 ± 4.1	16.5 ± 1.2	14.9 ± 0.7	48.6 ± 2.1	0.7 ± 0.3
	Current user (*n* = 2938)	301.5 ± 5.4	195.9 ± 3.6	18.9 ± 1.3	18.2 ± 0.7	66.9 ± 2.4	1.6 ± 0.7
	*p*-value (ANOVA)	< 0.01	< 0.01	0.20	< 0.01	< 0.01	0.32
Alcohol intake	0 ml/wk. (*n* = 6175)	218.9 ± 3.1	137.6 ± 2.4	19.5 ± 0.7	17.6 ± 0.5	39.5 ± 1.3	1.1 ± 0.3
	0.23–24.85 ml/wk. (*n* = 5486)	214.8 ± 2.6	149.6 ± 2.2	18.2 ± 0.6	14.6 ± 0.4	29.8 ± 1.1	0.5 ± 0.1
	24.86–71.69 ml/wk. (*n* = 5696)	239.0 ± 2.6	169.2 ± 2.2	17.2 ± 0.7	14.2 ± 0.4	35.3 ± 1.1	0.7 ± 0.2
	≥71.70 ml/wk. (*n* = 5817)	299.2 ± 3.6	211.1 ± 2.6	19.3 ± 0.8	16.7 ± 0.5	45.8 ± 1.5	1.8 ± 0.5
	*p*-value (ANOVA/trend)	< 0.01/< 0.01	< 0.01/< 0.01	0.05/0.60	< 0.01/0.17	< 0.01/< 0.01	0.02/0.13
Sleep	≥7 h/night (*n* = 8530)	221.8 ± 2.1	158.8 ± 1.7	17.3 ± 0.5	14.1 ± 0.3	29.0 ± 0.8	0.1 ± 0.0
	5–6 h/night (*n* = 9110)	263.3 ± 2.4	177.4 ± 2.0	18.8 ± 0.6	17.7 ± 0.4	45.4 ± 1.0	0.2 ± 0.1
	< 4 h/night (*n* = 918)	337.7 ± 11.9	199.4 ± 9.0	27.2 ± 2.6	21.2 ± 1.6	67.4 ± 4.7	6.4 ± 2.8
	*p*-value (ANOVA/trend)	< 0.01/< 0.01	< 0.01/< 0.01	< 0.01/< 0.01	< 0.01/< 0.01	< 0.01/< 0.01	< 0.01/< 0.01
Rank	Junior enlisted, E1–E4 (*n* = 1869)	197.4 ± 6.9	103.3 ± 3.8	20.3 ± 1.5	18.3 ± 0.9	53.4 ± 2.9	2.0 ± 1.1
	Mid enlisted, E4–E6 (*n* = 9891)	238.6 ± 2.3	148.0 ± 1.8	18.6 ± 0.6	16.6 ± 0.4	54.2 ± 1.1	1.2 ± 0.2
	Senior enlisted, E7–E9 (*n* = 3971)	270.2 ± 4.2	194.1 ± 3.2	18.0 ± 0.9	17.2 ± 0.6	39.6 ± 1.6	1.3 ± 0.5
	Warrant Officer (*n* = 537)	251.8 ± 8.2	190.0 ± 7.8	17.1 ± 2.0	12.8 ± 1.3	31.9 ± 3.1	0.1 ± 0.1
	Junior officer, O1–O3 (*n* = 3436)	233.6 ± 3.4	179.7 ± 2.8	16.6 ± 0.8	10.5 ± 0.5	26.3 ± 1.4	0.4 ± 0.1
	Senior officer, O4–O7 (*n* = 3471)	257.3 ± 3.1	206.1 ± 3.0	19.9 ± 0.8	16.4 ± 0.6	14.4 ± 0.9	0.4 ± 0.1
	*p*-value (ANOVA)	< 0.01	< 0.01	0.09	< 0.01	< 0.01	0.10
Occupational assignment group	Combat arms (*n* = 6451)	260.7 ± 3.0	183.8 ± 2.4	16.8 ± 0.7	14.8 ± 0.4	44.3 ± 1.3	1.0 ± 0.2
	Combat support (*n* = 10,424)	236.8 ± 2.4	158.7 ± 1.8	18.5 ± 0.6	15.9 ± 0.4	42.9 ± 1.0	0.8 ± 0.3
	Combat service support (*n* = 9132)	238.1 ± 2.6	164.1 ± 2.1	19.6 ± 0.6	16.5 ± 0.4	36.6 ± 1.2	1.3 ± 0.3
	*p*-value (ANOVA)	< 0.01	< 0.01	0.01	0.02	< 0.01	0.44
Service branch	Air Force (*n* = 8443)	215.0 ± 2.3	145.2 ± 1.7	17.7 ± 0.6	16.0 ± 0.4	35.9 ± 1.0	0.8 ± 0.3
	Army (*n* = 6907)	251.5 ± 2.9	173.6 ± 2.3	18.6 ± 0.7	15.5 ± 0.6	42.0 ± 1.2	1.3 ± 0.3
	Marine Corps (*n* = 2707)	269.7 ± 5.2	172.6 ± 3.9	16.7 ± 1.1	16.4 ± 0.5	63.7 ± 2.5	1.3 ± 0.4
	Navy (*n* = 5118)	263.9 ± 3.0	189.4 ± 2.6	20.7 ± 0.7	15.8 ± 0.2	36.3 ± 1.2	1.0 ± 0.2
	*p*-value (ANOVA)	< 0.01	< 0.01	< 0.01	0.36	< 0.01	0.58

*Abbreviation*: *ANOVA* analysis of variance

Men consumed more total caffeine than women due to a greater intake from coffee, soda, and energy drinks; women consumed more caffeine from tea. When total caffeine consumption was determined on a body weight basis, consumption was similar among male and female consumers (2.93 vs 2.85 mg/day/kg, men and women, respectively, *p* = 0.12). Caffeine consumption increased with age, largely accounted for by the increase from coffee, while caffeine consumption from energy drinks decreased with age. Caffeine consumed from tea and soda was greatest in the youngest and oldest age groups. Total caffeine consumption differed little by formal educational level, but caffeine from coffee increased with more formal education, while caffeine from soda, energy drinks, and gums/medications decreased with more formal education. White and Hispanic SMs consumed the most total caffeine, accounted for largely by coffee, soda, and energy beverages, while black SMs consumed the least total caffeine and had the least caffeine consumption from coffee, soda, and energy beverages. As BMI increased, so did total caffeine consumption, especially from coffee, soda, and energy drinks; caffeine from gums/medications was highest among the lowest BMI group.

As the amount of aerobic exercise increased, so did total caffeine consumption, accounted for largely by caffeine from coffee and energy drinks. Caffeine from soda decreased as aerobic exercise increased; caffeine from tea and gums/medications was highest in the group performing the most aerobic exercise. As the amount of resistance training increased, caffeine from coffee, tea, and soda tended to decrease, while caffeine from energy drinks increased. Among smokers and smokeless tobacco users, the pattern of caffeine consumption was similar: current and former users had the highest total caffeine consumption, accounted for largely by consumption from coffee, soda, and energy drinks. As alcohol intake increased, so did the total consumption of caffeine, accounted for by caffeine from coffee. Caffeine from tea, soda, energy drinks, and gums/medications was highest among non-alcohol users and those in the highest alcohol level. Consumption of caffeine from all sources increased as the amount of sleep decreased. The average ± standard deviation for self-reported sleep was 6.3 ± 1.4 h.

Consumption of total caffeine and caffeine from coffee increased with rank among enlisted personnel and officers, while consumption from energy drinks decreased with rank among enlisted and officers. Caffeine from soda decreased with rank among enlisted SMs but increased with rank among officers. SMs employed in combat arms occupations had the highest total consumption of caffeine and consumption from coffee and energy drinks, while those in combat service support occupations had the highest consumption from tea and soda. Marine Corps and Navy personnel had the highest total consumption of caffeine. Caffeine from coffee and tea was highest among Navy personnel, while caffeine from energy drinks was highest among Marine Corps personnel. Air Force personnel had the lowest total caffeine consumption and the lowest consumption from coffee and energy drinks.

### Characteristics independently associated with prevalence of caffeine consumers

Table 3 provides results of the multivariable logistic regression examining factors associated with the use of caffeinated products ≥1 time per week. The results are for six full models with all characteristics entered. About 91% (*n* = 24,324) of SMs had complete data on all variables and were included in each model.

**Table 3:** Characteristics associated with prevalence (≥1 time/week) of specific caffeine products among military personnel.^a^ Multivariable logistic regression, data are odds ratios with 95% confidence intervals in parenthesis

Variable	Strata	Caffeine Products Consumed ≥ 1 Time/Week [Odds ratio (95%confidence interval)]					
		Any Caffeine Product (Model 1)	Coffee (Model 2)	Tea (Model 3)	Soda (Model 4)	Energy Drink (Model 5)	Gum or Medication (Model 6)
Gender	Men	1.00	1.00	1.00	1.00	1.00	1.00
	Women	1.52 (1.34–1.72)	1.43 (1.31–1.56)	2.14 (1.97–2.32)	0.71 (0.66–0.77)	0.64 (0.58–0.71)	2.10 (1.78–2.49)
Age	18–24 years	1.00	1.00	1.00	1.00	1.00	1.00
	25–29 years	1.25 (1.10–1.44)	1.34 (1.21–1.49)	1.03 (0.92–1.14)	0.93 (0.84–1.02)	1.06 (0.96–1.17)	0.99 (0.77–1.26)
	30–39 years	1.69 (1.46–1.95)	1.59 (1.43–1.76)	1.08 (0.97–1.21)	1.05 (0.95–1.15)	1.01 (0.91–1.13)	1.34 (1.05–1.70)
	≥40 years	1.95 (1.61–2.38)	1.79 (1.56–2.05)	1.36 (1.19–1.55)	1.08 (0.96–1.23)	0.61 (0.53–0.70)	1.54 (1.15–2.05)
Formal Education	Some HS/HS grad	1.00	1.00	1.00	1.00	1.00	1.00
	Some college	1.07 (0.94–1.22)	1.27 (1.16–1.40)	1.13 (1.02–1.25)	0.84 (0.77–0.92)	0.93 (0.85–1.02)	0.93 (0.75–1.15)
	College degree	1.09 (0.92–1.30)	1.38 (1.22–1.56)	1.16 (1.03–1.31)	0.74 (0.66–0.83)	0.75 (0.67–0.85)	0.80 (0.62–1.04)
Race / Ethnicity	White	1.00	1.00	1.00	1.00	1.00	1.00
	Hispanic	0.64 (0.57–0.72)	0.87 (0.80–0.95)	0.88 (0.81–0.96)	0.86 (0.79–0.92)	0.80 (0.74–0.87)	0.75 (0.62–0.91)
	Back	0.27 (0.24–0.31)	0.36 (0.33–0.40)	1.08 (0.98–1.19)	0.71 (0.64–0.77)	0.47 (0.42–0.53)	0.84 (0.68–1.03)
	Other	0.65 (0.58–0.74)	0.90 (0.82–0.99)	1.35 (1.23–1.47)	0.88 (0.81–0.96)	0.78 (0.72–0.87)	0.74 (0.59–0.91)
Body mass index	< 25.0 kg/m^2^	1.00	1.00	1.00	1.00	1.00	1.00
	25.0–29.9 kg/m^2^	1.19 (1.08–1.30)	1.08 (1.01–1.16)	0.96 (0.90–1.03)	1.09 (1.02–1.15)	1.29 (1.20–1.38)	1.20 (1.02–1.40)
	≥30.0 kg/m^2^	1.46 (1.27–1.67)	1.08 (0.99–1.19)	1.00 (0.91–1.09)	1.43 (1.32–1.56)	1.58 (1.44–1.73)	1.61 (1.33–1.95)
Aerobic exercise duration	< 90 min/wk	1.00	1.00	1.00	1.00	1.00	1.00
	90–180 min/wk	1.11 (0.99–1.25)	1.07 (0.99–1.16)	1.12 (1.04–1.21)	1.05 (0.98–1.13)	1.01 (0.93–1.09)	0.93 (0.78–1.11)
	181–300 min/wk	1.07 (0.95–1.22)	1.05 (0.96–1.14)	1.14 (1.05–1.24)	1.01 (0.93–1.09)	1.00 (0.92–1.09)	0.86 (0.71–1.03)
	> 300 min/wk.	0.95 (0.84–1.08)	1.00 (0.91–1.09)	1.22 (1.12–1.33)	0.93 (0.86–1.01)	0.88 (0.81–0.96)	1.07 (0.88–1.29)
Resistance training duration	≤45 min/wk	1.00	1.00	1.00	1.00	1.00	1.00
	46–135 min/wk	1.08 (0.96–1.23)	1.16 (1.07–1.26)	0.99 (0.92–1.07)	0.85 (0.79–0.91)	1.03 (0.94–1.12)	1.00 (0.84–1.19)
	136–300 min/wk	1.06 (0.93–1.20)	1.22 (1.12–1.32)	0.93 (0.86–1.01)	0.63 (0.59–0.68)	1.11 (1.02–1.21)	0.97 (0.81–1.15)
	≥301 min/wk	0.75 (0.66–0.85)	0.98 (0.89–1.07)	0.74 (0.68–0.81)	0.49 (0.45–0.53)	1.30 (1.19–1.43)	0.88 (0.72–1.08)
Smoking	Never	1.00	1.00	1.00	1.00	1.00	1.00
	Former user	1.77 (1.54–2.05)	1.74 (1.59–1.90)	1.05 (0.97–1.14)	1.07 (1.00–1.16)	1.16 (1.07–1.26)	1.17 (0.98–1.38)
	Current user	2.00 (1.73–2.31)	1.68 (1.53–1.83)	1.09 (1.00–1.18)	1.45 (1.34–1.56)	1.57 (1.45–1.70)	1.13 (0.95–1.35)
Smokeless tobacco use	Never	1.00	1.00	1.00	1.00	1.00	1.00
	Former user	1.88 (1.48–2.39)	1.57 (1.37–1.79)	0.88 (0.78–0.98)	0.90 (0.82–1.00)	1.20 (1.08–1.33)	1.04 (0.82–1.32)
	Current user	1.58 (1.32–1.88)	1.44 (1.30–1.60)	0.91 (0.83–0.99)	1.07 (0.98–1.16)	1.52 (1.40–1.66)	0.99 (0.81–1.21)
Alcohol intake	0 ml/wk	1.00	1.00	1.00	1.00	1.00	1.00
	0.23–24.85 ml/wk.	2.16 (1.94–2.40)	1.85 (1.71–1.99)	1.38 (1.27–1.49)	1.19 (1.10–1.28)	1.08 (0.99–1.17)	0.98 (0.81–1.17)
	24.86–71.69 ml/wk.	3.39 (3.00–3.84)	2.36 (2.18–2.56)	1.51 (1.39–1.64)	1.39 (1.29–1.49)	1.39 (1.28–1.51)	1.15 (0.96–1.38)
	≥71.70 ml/wk	5.15 (4.44–5.98)	3.10 (2.84–3.38)	1.68 (1.54–1.82)	1.50 (1.39–1.62)	1.76 (1.62–1.92)	1.36 (1.14–1.63)
Rank	Junior enlisted, E1–E4	1.00	1.00	1.00	1.00	1.00	1.00
	Mid enlisted, E4–E6	1.13 (0.98–1.30)	1.07 (0.96–1.20)	0.99 (0.87–1.11)	1.02 (0.92–1.14)	1.03 (0.92–1.16)	1.13 (0.85–1.50)
	Senior enlisted, E7–E9	1.38 (1.12–1.69)	1.26 (1.08–1.46)	0.90 (0.77–1.04)	0.99 (0.86–1.13)	0.87 (0.75–1.01)	0.99 (0.70–1.38)
	Warrant officer, WO1-O5	1.86 (1.23–2.79)	1.62 (1.25–2.10)	0.88 (0.69–1.11)	0.87 (0.70–1.08)	0.81 (0.64–1.03)	0.93 (0.56–1.56)
	Junior officer, O1–O3	0.97 (0.79–1.20)	1.20 (1.03–1.40)	1.06 (0.90–1.24)	0.77 (0.67–0.89)	0.56 (0.48–0.66)	0.84 (0.58–1.22)
	Senior officer, O4–O7	1.36 (1.06–1.74)	1.27 (1.07–1.51)	0.98 (0.83–1.16)	0.94 (0.80–1.10)	0.41 (0.34–0.49)	0.78 (0.53–1.15)
Occupation assignment group	Combat arms	1.00	1.00	1.00	1.00	1.00	1.00
	Combat support	1.01 (0.90–1.13)	0.92 (0.85–1.00)	1.05 (0.97–1.13)	1.04 (0.97–1.12)	0.96 (0.89–1.04)	1.09 (0.92–1.29)
	Combat service support	1.00 (0.89–1.12)	0.96 (0.89–1.04)	1.00 (0.93–1.08)	1.11 (1.03–1.19)	0.92 (0.85–1.00)	1.12 (0.95–1.33)
Service branch	Air Force	1.00	1.00	1.00	1.00	1.00	1.00
	Army	1.02 (0.92–1.14)	1.03 (0.95–1.12)	0.93 (0.87–1.00)	0.93 (0.87–0.99)	1.11 (1.03–1.20)	1.18 (1.00–1.40)
	Marine Corps	0.98 (0.85–1.13)	1.09 (0.98–1.20)	0.90 (0.81–0.99)	0.88 (0.80–0.96)	1.26 (1.15–1.39)	1.03 (0.82–1.30)
	Navy	1.03 (0.91–1.16)	1.23 (1.13–1.33)	1.10 (1.02–1.19)	0.90 (0.83–0.97)	0.90 (0.82–0.98)	1.18 (0.99–1.40)

*Abbreviation*: *HS* high school

^a^All six models are adjusted for gender, age, formal education, race/ethnicity, body mass index, aerobic exercise duration, resistance exercise duration, smoking, smokeless tobacco use, alcohol intake, rank, occupational assignment group, and service branch

Characteristics associated with higher overall caffeine use included female gender, older age, white race/ethnicity, higher BMI, less resistance training, current or former tobacco use, higher alcohol intake, and higher enlisted or officer rank. Higher coffee use was associated with female gender, older age, higher formal education, white race/ethnicity, higher BMI, former or current tobacco use, higher alcohol intake, higher enlisted or officer rank, and service in the Navy (compared to the Air Force). Higher use of tea was associated with female gender, older age, more formal education, other race/ethnicity (compared to whites), white race/ethnicity (compared to Hispanics), more aerobic exercise, less resistance training, current smoking, never using smokeless tobacco, higher alcohol intake, and service in the Navy (compared to the Air Force) or Air Force (compared to the Marine Corps). Higher use of soda was associated with male gender, less formal education, white race/ethnicity, higher BMI, less resistance training, current or former smoking, higher alcohol consumption, junior enlisted status (compared to junior officer status), and service in the Air Force (compared to all other services). Higher use of energy drinks was associated with male gender, younger age, less formal education, white race/ethnicity, higher BMI, more resistance training, current or former tobacco use, higher alcohol consumption, lower enlisted rank (compared to officers), and service in the Army or Marine Corps (compared to the Air Force) or in the Air Force (compared to the Navy). Higher use of caffeinated gums/medication was independently associated with female gender, older age, white and black race/ethnicity, higher BMI, higher alcohol intake, and service in the Army (compared to the Air Force).

### Prevalence and characteristics of high caffeine consumers

The proportion of high caffeine consumers (≥400 mg/day) was 15.9% (17.1% of men and 8.9% of women), and the proportion with an overall consumption ≥300 mg/day was 27.3% (28.8% of men and 17.5% of women). The types of products ingested by the high caffeine consumers were similar to those of the entire cohort: coffee, teas, sodas, energy drinks, and gums/medications accounted for 68, 7, 5, 19, and 1% of caffeine consumption, respectively.

Table 4 compares high caffeine consumers (≥400 mg/day) to lower consumers (≤400 mg/day) on their demographic, lifestyle and military characteristics. In univariable analyses, higher caffeine use was associated with male gender, older age, less formal education, white race/ethnicity, higher BMI, more aerobic exercise, less resistance training, tobacco use or former use, higher alcohol intake, less sleep, higher enlisted or officer rank, combat arms occupations, and service in the Army, Marine Corps, or Navy (compared to the Air Force). About 91% of caffeine consumers (*n* = 21,443) had complete data for the multivariate model. In the multivariable analyses with all characteristics included, most of the relationships found in the univariate analyses were retained, although somewhat attenuated; rank and occupational assignment group were no longer significant.

**Table 4:** Comparison of Lower (< 400 mg/day) and High (≥400 mg/day) Caffeine Consumers

Variable	Strata	Prevalence of High Caffeine Consumers (% ± SE)	Univariable Analysis		Multivariable Analysis	
			n	Odds Ratio (95%CI)	n	Odds Ratio (95%CI)
Gender	Men	17.1 ± 0.3	20,040	1.00	18,537	1.00
	Women	8.7 ± 0.5	3135	0.46 (0.41–0.53)	2906	0.68 (0,.59–0.78)
Age	18–24 years	12.6 ± 0.6	3615	1.00	3341	1.00
	25–29 years	13.6 ± 0.5	4746	1.09 (0.96–1.24)	4435	1.12 (0.95–1.30)
	30–39 years	17.3 ± 0.4	9875	1.45 (1.30–1.62)	9189	1.46 (1.25–1.70)
	≥40 years	18.0 ± 0.6	4821	1.53 (1.35–1.73)	4478	1.54 (1.25–1.85)
Formal Education	Some HS/HS grad	17.8 ± 0.7	3099	1.00	2831	1.00
	Some college	16.2 ± 0.4	9836	0.89 (0.80–0.99)	9109	0.87 (0.77–0.99)
	College degree	15.1 ± 0.4	10,234	0.82 (0.74–0.91)	9503	0.82 (0.70–0.96)
Race / Ethnicity	White	18.1 ± 0.3	14,827	1.00	13,751	1.00
	Hispanic	13.9 ± 0.6	3530	0.73 (0.66–0.81)	3280	0.75 (0.66–0.84)
	Back	7.2 ± 0.6	2.140	0.35 (0.30–0.42)	1947	0.36 (0.30–0.44)
	Other	13.6 ± 0.7	2678	0.71 (0.63–0.80)	2465	0.73 (0.64–0.83)
Body mass index	< 25.0 kg/m^2^	12.0 ± 0.4	6629	1.00	6282	1.00
	25.0–29.9 kg/m^2^	16.8 ± 0.3	12,163	1.48 (1.36–1.62)	11,462	1.28 (1.16–1.40)
	≥30.0 kg/m^2^	19.3 ± 0.6	3956	1.76 (1.58–1.96)	3699	1.48 (1.32–1.67)
Aerobic exercise duration	< 90 min/wk	15.1 ± 0.5	6120	1.00	5555	1.00
	90–180 min/wk	15.4 ± 0.5	6560	1.02 (0.92–1.12)	6100	1.03 (0.93–1.15)
	181–300 min/wk	16.6 ± 0.5	5218	1.12 (1.01–1.23)	4879	1.14 (1.02–1.28)
	> 300 min/wk.	16.9 ± 0.5	5277	1.14 (1.03–1.26)	4909	1.24 (1.11–1.40)
Resistance training duration	≤45 min/wk	17.1 ± 0.5	6650	1.00	5965	1.00
	46–135 min/wk	15.5 ± 0.5	5636	0.89 (0.81–0.98)	5257	0.90 (0.81–1.00)
	136–300 min/wk	15.3 ± 0.5	5859	0.87 (0.79–0.96)	5477	0.83 (0.75–0.93)
	≥301 min/wk	15.5 ± 0.5	5030	0.89 (0.80–0.98)	4744	0.80 (0.71–0.90)
Smoking	Never	12.2 ± 0.3	14,177	1.00	13,359	1.00
	Former user	20.5 ± 0.6	4475	1.86 (1.70–2.03)	4117	1.42 (1.28–1.58)
	Current user	23.5 ± 0.7	4222	2.21 (2.03–2.41)	3967	1.79 (1.62–1.97)
Smokeless tobacco use	Never	13.7 ± 0.3	17,597	1.00	16,767	1.00
	Former user	23.2 ± 1.0	1959	1.90 (1.70–2.13)	1861	1.25 (1.10–1.42)
	Current user	23.7 ± 0.8	2938	1.95 (1.78–2.15)	2815	1.28 (1.15–1.42)
Alcohol intake	0 ml/wk	14.3 ± 0.5	6175	1.00	5533	1.00
	0.23–24.85 ml/wk.	11.8 ± 0.4	5486	0.80 (0.72–0.89)	5099	0.79 (0.70–0.89)
	24.86–71.69 ml/wk.	14.3 ± 0.5	5696	1.00 (0.90–1.11)	5353	0.87 (0.78–0.97)
	≥71.70 ml/wk	23.3 ± 0.6	5817	1.83 (1.66–2.01)	5458	1.42 (1.28–1.58)
Sleep	≥7 h/night	12.7 ± 0.4	8530	1.00		
	5–6 h/night	19.3 ± 0.4	9110	1.65 (1.52–1.79)	a	a
	< 4 h/night	25.1 ± 1.4	918	2.30 (1.95–2.70)		
Rank	Junior enlisted, E1–E4	12.0 ± 0.8	1869	1.00	1735	1.00
	Mid enlisted, E4–E6	15.8 ± 0.4	9891	1.37 (1.18–1.60)	9129	1.01 (0.84–1.21)
	Senior enlisted, E7–E9	19.3 ± 0..6	3971	1.76 (1,50–2.07)	3653	1.05 (0.85–1.30)
	Warrant officer, WO1-O5	17.1 ± 1.6	537	1.52 (1.17–1.98)	501	0.84 (0.61–1.16)
	Junior officer, O1–O3	14.1 ± 0.6	3436	1.20 (1.01–1.42)	3266	0.91 (0.72–1.15)
	Senior officer, O4–O7	16.3 ± 0.6	3471	1.43 (1.21–1.69)	3159	0.94 (0.73–1.20)
Occupation assignment group	Combat arms	18.5 ± 0.5	5742	1.00	5460	1.00
	Combat support	15.3 ± 0.4	8992	0.79 (0.73–0.87)	8567	0.91 (0.83–1.00)
	Combat service support	14.9 ± 0.4	7881	0.77 (0.70–0.84)	7416	0.93 (0.84–1.02)
Service branch	Air Force	12.0 ± 0.4	8443	1.00	7777	1.00
	Army	17.0 ± 0.5	6907	1.50 (1.37–1.64)	6494	1.28 (1.15–1.42)
	Marine Corps	19.9 ± 0.8	2707	1.82 (1.62–2.04)	2534	1.58 (1.38–1.79)
	Navy	18.9 ± 0.6	5118	1.71 (1.56–1.88)	4638	1.52 (1.37–1.69)

^a^Not inclued in multivariable analysis because of amount of missing data

*Abbreviation*: *HS* high school

### Caffeine consumption by age and sex

**Fig. 1:**
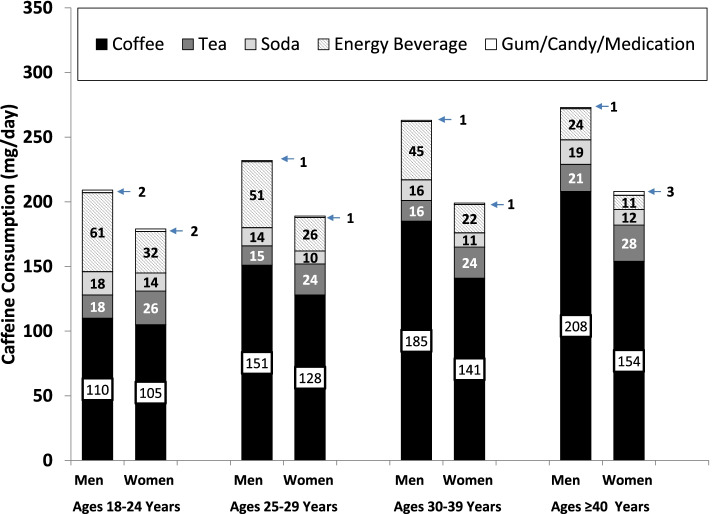
Daily average consumption of caffeinated substances by gender and age

Figure 1 presents daily caffeine consumption (mg/day) from all types of caffeinated products by age and sex. As age increased, there was a significant linear trend for increasing consumption of any caffeine and caffeine from coffee among both men and women (*p* < 0.01, both sexes). In contrast, there was a significant linear trend for less consumption of energy drinks as age increased for both men and women (*p* < 0.01, both sexes). While there was a significant linear trend of increased caffeine consumption from tea over age among men (*p* = 0.02), there was no such trend among women (*p* = 0.42). There were no significant linear trends over age for soda (men *p* = 0.07, women *p* = 0.48) or for gums/medications (men *p* = 0.13, women *p* = 0.82).

## Discussion

This very large (*n* = 26,680), comprehensive assessment of SM caffeine consumption found 87% of SMs consumed caffeinated products, with an average estimated consumption of 243 mg/day for consumers. Men consumed more caffeine than women, but when adjusted for body weight, consumption was similar by gender. Coffee was the most frequently consumed beverage, followed in descending order of prevalence by soda, tea, energy drinks, and gums/candies/medications. By total caffeine consumption (mg/day) and in desending order coffee, energy drinks, tea, soda, and gums/medications were the most often used. Consuming any caffeinated product was independently associated with female gender, older age, white race/ethnicity, higher BMI, less resistance training, current or former tobacco use, higher alcohol intake, and higher enlisted or officer rank. Higher energy drink prevalence was associated with male gender, younger age, less formal education, white race/ethnicity, higher BMI, more resistance training, current or former tobacco use, higher alcohol consumption, lower enlisted rank (compared to officers), and service in the Army or Marine Corps (compared to the Air Force) or in the Air Force (compared to the Navy).

It is well documented that the civilian and military populations are generally aware of the effects of caffeine on human cognitive and physical performance. Surveys of SMs and college students found they consume caffeine-containing products for several reasons related to the performance benefits of caffeine [19, 30, 31]. Furthermore, SMs assigned to units in Afghanistan and likely to be engaged in combat consumed higher levels of caffeine than SMs at their home bases. Caffeine use by these SMs was higher among those reporting difficulty remaining awake during guard duty, poor sleeping conditions, and sleep disruptions during nighttime operations [18]. In addition, a survey of active duty Army aviators found they consumed more caffeine than their peers in non-aviation units, especially to enhance performance degraded due to insufficient sleep and very disruptive work schedules [19].

US Department of Defense laboratories and their international collaborators have conducted multiple studies designed to simulate military operations demonstrating the cognitive and physical benefits of caffeine consumption by military personnel [32–34]. The Department of Defense recognizes the ability of caffeine to enhance cognitive performance and provides it in rations, when necessary, with appropriate labeling to inform SMs of the presence and effects of caffeine [35].

### Prevalence of caffeine consumers and daily caffeine consumption

Previous studies have been conducted on the prevalence of caffeine consumers and daily consumption among Air Force [22], Army [20], and Navy/Marine Corps [21] personnel. All of these studies [20–22] used a slightly different questionnaire but the same definitions for caffeine sources. The Air Force [22] and Army [20] studies used a convenience sampling technique involving volunteers in face-to-face administrations at installations across the US and overseas, and the Navy and Marine Corps study [21] identified a random sample and asked for volunteers by postal letter and e-mail. The present study was quite similar to the Navy/Marine Corps study [21] in that a random sample of SMs were studied, but the questionnaire differed from that of previous studies [20–22]. Those studies listed not only generic sources of caffeine (e.g., coffee, tea, soft drinks), as in the present study, but specific products (e.g., Dr. Pepper soda, Monster energy drink, No Doz Gum) as well. Given these differences in study design, Table 5 compares caffeine use prevalence and daily consumption among the military services in the current and past studies. Estimates of the prevalence of caffeine consumers for any caffeinated product (≥ 1/week) were similar across all studies. With regard to individual caffeinated products, the previous Army and Navy/Marine Corps studies [20, 21] found the highest prevalence of consumers for coffee, but Air Force personnel were unique in that cola was the most ingested product, with coffee ranking second [22]. The current study found that in all services, coffee was the product consumed most often. Daily caffeine consumption estimates were similar for Air Force personnel in the current and past [22] investigations, but estimates for Army, Navy, and Marine Corps personnel were 38% lower, 21% higher, and 16% higher, respectively [20, 21]. Differences in estimation of caffeine consumption from individual products in past studies [20–22] versus estimates from generic types (coffee, tea, soda) in the current study likely accounted for these differences. Most past studies [20, 21] and the current one agree in that SMs in all services consumed the most total caffeine (mg/day) from coffee, with energy drinks ranking second.

**Table 5:** Comparison of studies on prevalence and amount of caffeine consumption in military services^a^

Measure	Study	Air Force	Army	Marine Corps	Navy
Prevalence (%)^b^	Previous^c^	84	82	86	88
	Current	86	87	85	89
Daily Consumption (mg/day)	Previous^c^	212	347	232	217
	Current	215	252	270	264

^a^Caffeine consumers only

^b^Caffeine prevalence is defined as use ≥1 time/week

^c^Previous studies include those of the Army [20], Air Force [22], and Navy/Marine Corps [21]

There have also been several studies of the prevalence of caffeine consumers and consumption in the military of other countries, although all studies used convenience samples and many were conducted over a decade ago. Among British soldiers deployed to Iraq in 2009, and Afghanistan in 2010, 89 and 92%, respectively, reported consuming a caffeinated product [36]. In 2010–2011, 42% of United Kingdom-based British soldiers reported using energy drinks and 8% caffeine tablets [37]. Among Australian soldiers, 71% reported using caffeinated products and 28% reporting energy drink consumption [38]. New 19-yr old Danish conscripts surveyed in 2001–2006 reported consuming an average of 199 mg/day from coffee, tea, soda, and foods [39]. These data indicate that British soldiers and US SMs have a similar prevalence of caffeine use, but Australian soldiers appear to have a lower use prevalence. Nonetheless, Australian soldiers have a very similar prevalence of energy drink use compared to the US SMs, but British soldiers report much higher use. Overall caffeine consumption in comparably aged US military personnel appears similar to that of Danish conscripts.

Several population-based estimates of caffeine consumption in adult Americans based on very large population samples using state-of-the-art dietary intake procedures are available. NHANES caffeine intake [1, 13, 15] was calculated based on 24-h dietary recalls in 2001–2012. Estimated caffeine use prevalence in adults (> 19 years) was 89% for men and 89% for women [1]. Caffeine consumption estimates for consumers of caffeine varied from 189 to 211 mg/day for men and 149 to 161 mg/day for women [1, 13, 15]. The Kantar Worldpanel Beverage Consumption Panel obtained data on US consumers from an online, 7-day beverage consumption record and found ~ 90% of individuals ≥18 years of age consumed caffeinated beverages, with average caffeine consumption equal to about 200 mg/day among caffeine consumers [2]. The prevalence of caffeine consumers in these population-based studies were similar to the 87% observed in SMs (≥1 week), while the average consumption in SMs of 251 and 195 mg/day for males and females, respectively, was somewhat higher than in the civilian population.

At least three other surveys [20–22] of the individual branches of service have observed caffeine-intake levels similar to those reported here and higher than those in the civilian population. The extensive and unique demands of military service may be a factor that explains the difference in caffeine intake in military versus civilian personnel. Differences in the methods and the demographic characteristics of the samples used in civilian studies and the current investigation must also be considered when interpreting these differences. For example, active duty SMs are younger, fully employed, and sleep somewhat less than the general population [16].

Energy drink prevalence (≥ 1 time/week) was 29% in the present study and varied from 21 to 39% in the previous military studies [20–22, 40–42]. Various studies of the prevalence of energy drink use among US college students found that 39% reported consuming in the past week [43], 36% within the past 2 weeks [44] and 36% within the past year [30]. Data from several NHANES cycles indicated that prevalence of daily consumption of energy drinks among adults has increased from 2003 to 2016 [10]. With regard to caffeine consumption, the current study found that 17% of the total caffeine was consumed from energy drinks. Data from NHANES suggested only 1–2% of total caffeine consumed by Americans was from energy drinks [1, 13], but a study of a convenience sample of geographically dispersed college students in the US found 22% of their total caffeine consumption was from energy drinks [30]. In summary, the prevalence of energy drink consumption by SMs, and the proportion of total caffeine consumption from energy drinks by SMs, are similar to those of college students— despite the generally older age of SMs—and much higher than those of the general US population.

### Characteristics associated with caffeine use

In the univariate analysis, there was little gender difference in the prevalence of use for any caffeinated products and for coffee. In the multivariate analysis, however, women had greater odds of use than men. This was primarily due to the confounding influence of alcohol consumption in the statistical models, although smoking and smokeless tobacco also had minor effects. Caffeine consumption increased as alcohol intake increased, or if individuals were tobacco users; men were more likely to be higher alcohol consumers or tobacco users. The strength of the association between caffeine use and alcohol and tobacco use was stronger in men than women and this reduced the effect of male gender alone. This reduced effect of male gender allowed female gender to become highly significant. In statistical terms, alcohol or tobacco use accounted for a larger proportion of the odds ratio for the effect of sex on caffeine use in men than in women. Because of this, the odds of using caffeine became lower in men than in women. Dividing the higher odds of caffeine use in women by the lower odds of caffeine use in men resulted in the larger odds ratio for women for any caffeinated product and coffee. If alcohol consumption, smoking, and smokeless tobacco use were not included in models 1 and 2 (Table 3) the odds ratios (95% confidence intervals) for women (compared to men) were 1.02 (0.92–1.14) and 1.02 (0.94–1.11), respectively.

In agreement with the current study, others [1, 12, 13, 20, 21] have reported that men consumed more caffeine than women. Nonetheless, this study and others [12, 21, 22] found that when caffeine consumption was determined on a per kg body weight basis, men and women consumed similar amounts. Although coffee was the major source of caffeine for both men and women, female SMs consumed more caffeine from tea while male SMs consumed more caffeine from soda and energy drinks. Acute caffeine consumption modestly affects moods such as vigor and fatigue as well as hemodynamic measures (e.g. blood pressure, cardiac output) in men and women [45–47], although cardiovascular effects are more likely to be observed at higher doses. Both men and women report consuming caffeinated products to provide behavioral benefits such as increased alertness [19, 30, 31].

Investigations involving representative civilian [1, 2, 12, 13, 27] and military [21, 22] samples reported that overall use and/or amount of caffeine consumption increased with age, although prevalence of use and/or caffeine amounts decline at the highest age groups in civilian studies (generally > 65 years) [1, 2, 12, 13, 27]. Also in general agreement with past military studies [20–22], the current study found that coffee consumption accounted for most of the caffeine ingested in all age groups, but younger (< 40 years) individuals consumed over twice as much caffeine from energy drinks as older (≥40 years) individuals (46 vs 22 mg/day, *p* < 0.01) and were almost twice as likely to consume energy drinks (33 vs 17%, *p* < 0.01). Energy drinks were introduced into the American market in 1997 [48], and their advertising was targeted to teenagers and individuals in 18- to 34-year-olds [49]. This advertising may have influenced energy drink consumption in the younger age groups in the current study.

Other civilian [13, 15] and military [20–22] studies have reported that compared to whites, blacks have a lower prevalence of caffeine use and a lower total caffeine consumption, accounted for largely by less coffee consumption [20–22, 26]. There are race/ethnic differences in dietary intake [50, 51], and some of these differences appear to be partly accounted for by educational level and income [51, 52]. In the current study, differences between black and white SMs in caffeine and coffee use prevalence remained after controlling for formal educational level, rank (a surrogate for income), and other factors, in agreement with past military studies [21, 22]. The reasons for the race/ethnic differences are likely complex and may be different in the military compared to the general population.

In agreement with other investigations [20, 21, 30], the current study found no systematic association between weekly aerobic exercise duration and caffeine use prevalence. One study of Air Force personnel [22] found that the prevalence of caffeine consumers decreased with increased aerobic activity duration; in the current study, when Air Force personnel were considered separately, this relationship was not duplicated (data not shown). For resistance training, both univariate and multivariable analysis showed the lowest caffeine use prevalence in the group exercising the most with little difference among the other groups, in general agreement with most other military studies [21, 22]. One study which separated Army personnel into those who performed weight training and those who did not found that trainers had higher overall use prevalence [20], also in agreement with the current study. Previous military studies have shown that dietary supplement use was strongly associated with increasing resistance training duration [21, 23]. Many dietary supplements contain caffeine, and the caffeine content of some of these can be very high [53]. Accurately determining the caffeine content of dietary supplements is difficult because manufactures are not required to list the amount of caffeine on their supplement facts labels, amounts are usually not available on company websites, and if the ingredients are proprietary, the manufacturer is not required to list caffeine at all [53]. It is possible that SMs involved in large amounts of resistance training consumed less caffeine from beverages to avoid adverse effects resulting from high dosages of caffeine in their dietary supplements. Overall, the current data and previous investigations suggest little relationship between aerobic exercise duration and caffeine use prevalence, but for resistance training there appears to be a bimodal relationship such that those performing the least or the most training have lower use prevalence than those performing moderate amounts of training.

Current or former tobacco use (smoking or smokeless tobacco) was associated with a higher use and higher intake of caffeine, especially for coffee and energy drinks, in both univariate and multivariable analyses. Although associations with smokeless tobacco have not been previously investigated, associations between caffeine use prevalence and smoking have repeatedly been reported in both military [20, 22] and civilian populations [14, 15, 54–60]. Smoking accelerates caffeine metabolism and reduces its half-life [61, 62] suggesting that smokers consume more caffeine to achieve stimulatory effects. In addition, both caffeine and smoking increase dopaminergic activity in different brain regions, and the two substances may be used concurrently to potentiate stimulation [63].

Another lifestyle factor strongly associated with prevalence of caffeine consumers and caffeine consumption was alcohol intake. In both univariate and multivariable analyses, use of caffeinated products of all types increased in a dose-response manner as alcohol consumption increased. The amount of caffeine consumed from coffee and energy drinks increased as alcohol intake increased. Similar relationships have been found in other studies for coffee [14, 21, 58, 64], energy drinks [21, 28, 65, 66], and overall caffeine use [15, 21, 28]. Studies of monozygotic and dizygotic twins suggested that there was a common genetic factor underlying this association, but environmental influences still seemed to contribute to the variance in caffeine consumption [67–69]. A recent study based on variations in single nucleotide polymorphisms support that the genes underlying the use of both coffee and alcohol were heritable [70]; however, two-sample Mendelian randomization suggested there was no causal association between coffee consumption and alcohol consumption [70, 71]. Psychosocial factors may play a role in this association since studies have consistently shown that higher levels of sensation–seeking behaviors are associated with both higher caffeine and alcohol use [72–74].

In the current study, SMs who reported less daily sleep consumed more caffeine for all sources, in agreement with past military [21, 22, 75] and civilian [76] studies. Military personnel sleep less than civilian populations [16, 17] and averaged 6.3 h in current study, less than the recommended ≥7 h/night [77]. Military training and operations can occur at any time of the day, can extend continuously for many days, and can involve substantial loss of sleep. Caffeine can increase alertness due to its ability to block central adenosine receptors [78]; when ingested in sufficient dosages, it can reduce sleep duration [79], and it improves cognitive performance, especially vigilance [80–82].

### High caffeine consumers

The estimated average daily caffeine consumption of military personnel who are regular caffeine consumers was well below the levels that are widely recognized as safe: 400 mg/day for men and 300 mg/day for women of reproductive age [5–7]. Nonetheless, the present study found that caffeine consumption of 17% of men and 9% of women exceeded 400 mg/day, and that of 18% of women exceeded 300 mg/day. These proportions are similar to those found in past military studies [21, 22]. Some individuals may be able to consume higher amounts of caffeine without adverse effects, although this cannot be determined from the current data. A genetic polymorphism allows some individuals to metabolize (N^3^-demethylation) caffeine in the liver more rapidly than others, and another polymorphism may be associated with higher caffeine tolerance and consumption [83–85].

Interestingly, the proportions of caffeine consumed from various dietary sources were very similar for the entire cohort and high caffeine consumers, suggesting high consumers just ingested a larger volume of these products. High caffeine consumers also shared many of the demographic and lifestyle characteristics of the entire cohort, except that they were almost twice as likely to be men, had less formal education, and were less likely to serve in the Air Force. Women have greater health awareness in that they are more likely to seek medical care [86–88] and make behavioral changes to improve health [89–91] that could moderate caffeine consumption. Individuals who have achieved higher education levels are generally more proactive, health conscious, prone to engage in health promoting behaviors, and likely to explore multiple channels of information related to their health [92–95], that could also be associated with management of caffeine consumption.

### Strengths and limitations

A major strength of this study was recruitment of a very large, stratified random sample of SMs who answered a standard set of questions on their consumption of specific caffeinated products. With a few exceptions, the data largely confirm results of past investigations of caffeine prevalence and consumption involving smaller studies of separate military services, using largely convenience samples [20–22]. Nonetheless, there are several limitations to the current analyses, most of which relate to difficulty in estimating daily caffeine consumption. First, all data were self-reported, and the usual shortcomings associated with this method, including recall bias, social desirability, errors in self-observation, and inadequate recall, apply [96, 97]. These biases could account for errors in reporting serving sizes and how many times per week SMs used caffeinated products and, as a consequence, errors in estimating caffeine consumption. Second, caffeine data for this study were obtained from beverages and gums/medications; we purposely did not assess caffeine intake from food sources as beverages account for 98% of caffeine consumption [1]. Third, caffeine contents of products were estimated based on standardized values of each type of caffeinated product. Specific products can differ in caffeine content [29, 98–100]. Fourth, the questionnaire used in this study was not validated against other measures of caffeine consumption such as plasma caffeine levels or beverage records. Fifth, caffeine from dietary supplements was not assessed and it is known that SMs use a larger number of dietary supplements [23]. This likely resulted in an underestimate of total caffeine consumption. Thus, this study is focused on caffeine consumption from commonly consumed caffeine sources exclusive of dietary supplements. Finally, a large number of statistical tests examining relationships between caffeine prevalence and consumption and the demographic, lifestyle, and military characteristics were conducted, thus increasing the probability of Type 1 errors.

## Conclusions

Among all military personnel surveyed, 87% reported using caffeinated products ≥1 time/week, with male and female consumers ingesting (mean ± SE) 251 ± 2 and 195 ± 3 mg/day, respectively. The prevalence of caffeine consumption by military personnel was similar to that reported in NHANES data, but total caffeine consumption was higher. Compared to civilians, SMs may consume more caffeine to enhance their cognitive and physical performance due to the intense occupational demands of their profession. The most commonly consumed caffeinated products (% users) were coffee (68%), soda (42%), tea (29%), and energy drinks (29%). Coffee, tea, soda, energy drinks, and gums/medications accounted for 69, 8, 6, 17, and > 1% of total caffeine consumption, respectively. The prevalence of energy drinks consumption and amount of caffeine ingested from energy drinks was about twice as high among those < 40 years of age compared to those ≥40 years of age. Characteristics associated with caffeine use in SMs were generally similar to those observed in investigations of civilians.

## Data Availability

The datasets generated and/or analyzed during the current study are not publicly available due to US government restrictions, but are available from the corresponding author on reasonable request.

## Abbreviations

ANOVA: Analysis of variance
BMI: Body mass index
NHANES: National Health and Nutrition Examination Survey
SM: Service member
SE: Standard error
US: United States
